# Importance of Parameter Settings on the Benefits of Robot-to-Robot Learning in Evolutionary Robotics

**DOI:** 10.3389/frobt.2019.00010

**Published:** 2019-03-04

**Authors:** Jacqueline Heinerman, Evert Haasdijk, A. E. Eiben

**Affiliations:** Department of Computer Science, Vrije Universiteit Amsterdam, Amsterdam, Netherlands

**Keywords:** social learning, robot-to-robot learning, evolutionary robotics, parameter tuning, neural networks, evolutionary algorithms

## Abstract

Robot-to-robot learning, a specific case of social learning in robotics, enables multiple robots to share learned skills while completing a task. The literature offers various statements of its benefits. Robots using this type of social learning can reach a higher performance, an increased learning speed, or both, compared to robots using individual learning only. No general explanation has been advanced for the difference in observations, which make the results highly dependent on the particular system and parameter setting. In this paper, we perform a detailed analysis into the effects of robot-to-robot learning. As a result, we show that this type of social learning can reduce the sensitivity of the learning process to the choice of parameters in two ways. First, robot-to-robot learning can reduce the number of bad performing individuals in the population. Second, robot-to-robot learning can increase the chance of having a successful run, where success is defined as the presence of a high performing individual. Additionally, we show that robot-to-robot learning results in an increased learning speed for almost all parameter settings. Our results indicate that robot-to-robot learning is a powerful mechanism which leads to benefits in both performance and learning speed.

## 1. Introduction

The widely used definition of social learning reflects animal behavior: social learning is learning through observation of conspecifics. Considering humans the definition can be extended: social learning is learning through observation of conspecifics or transferring knowledge through language. That is, the ability to use language offers a new method, a second tool in the toolbox of social learning. Regarding robots, we can add a third tool to this toolbox based on the ability to transfer robot controllers directly from one robot to another. (In common parlance this would be the robotic equivalent of telepathy.) Thus, the definition of social learning can be broadened again. If robots are concerned then social learning is learning through observation of conspecifics or transferring knowledge through language or direct exchange of (parts of) controllers.

In the current paper we focus on the third option for robots, the direct exchange of controllers, that is a special case of social learning that is only available for robots. To emphasize this we usethe term robot-to-robot learning.

Consider a collective of autonomous robots in an environment that is not well understood or modeled at design time. It is not possible to develop and validate adequate robot controllers without a thorough understanding of the environment, so the robots need to be able to adapt their behavior to suit. It is preferable that the robots are capable of learning autonomously, without the need for centraloversight: such a centralized scheme implies a single point of failure in the combined system.

To illustrate our concept of adaptation, consider the controller of a robot as a process that maps inputs, read from the robot's sensors and internal states to outputs, typically actuator and state settings. Learning can then be defined as any change to the mapping between inputs and outputs, cf. (Haasdijk et al., 2013). In such a setting, the robots can learn individually, e.g., by encapsulating a self-sufficient learning algorithm within each robot, and they can learn collectively by sharing knowledge, called robot-to-robot learning.

Robot-to-robot learning in a robotic collective has been studied for different machine learning implementations such as Reinforcement Learning (e.g., Sutton and Barto, 1998; Zhang et al., 2010; Noble and Franks, 2012; Wiering and van Otterlo, 2012) and Evolutionary Algorithms (EAs) (e.g., Pugh and Martinoli, 2009; Eiben and Smith, 2015a,b). In this paper, we consider evolutionary algorithms applied to robotics, i.e., Evolutionary Robotics (Nolfi and Floreano, 2000).

There is ample evidence that set-ups, where robots can share knowledge, outperform otherwise equivalent set-ups where robots learn in isolation.When robots share knowledge, they achieve better performance and/or the learning curve is steeper (Usui and Arita, 2003; Curran and ORiordan, 2007; Perez et al., 2008; Pugh and Martinoli, 2009; Garca-Sanchez et al., 2012; Miikkulainen et al., 2012; Tansey et al., 2012; Heinerman et al., 2015a,b; Jolley et al., 2016). A higher overall performance can be observed when there is a quality or diversity assessment before the knowledge is sent or incorporated (Huijsman et al., 2011; Garca-Sanchez et al., 2012; Heinerman et al., 2015b). Evidence by Huijsman et al. (2011) and Silva et al. (2015) show that robot-to-robot learning can linearly decrease learning time, e.g., the fitness measure that four robots can reach in 2 h can be reached by eight robots in 1 h when they learn socially. Although there is evidence that robot-to-robot learning can increase performance and/or learning speed, no general explanation has been advanced for the difference in observations. As an example, Usui and Arita (2003) showed that the speed of adaptation (not the finalperformance level) improves in hybrid set-ups compared to purely distributed ones, but that this improvement depends on the size of the encapsulated population. The authors of Pugh and Martinoli (2009) also varied the population size but did not find a significant effect of the population size on the performance. Because of these contradicting results, it is difficult to generalize on the benefits of robot-to-robot learning. This makes the results highly dependent on the particular system and parameter settings.

In our research, we increase our understanding of robot-to-robot learning by studying the dependence of the parameter settings on the benefits of robot-to-robot learning within one system. First, we observe the performance and learning speed of anindividual learning robot, learning a foraging task, when using different parameter settings. Then we compare the performance and learning speed with a setup where we enable the exchange of knowledge. This analysis enables us to observe when robot-to-robot learning leads to particular benefits. As a result, it brings us closer to understanding how robot-to-robot learning can improve performance and/or learning speed.

In previous work (Heinerman et al., 2017), we have shown that the observed advantages of robot-to-robot learning depend on the quality of the parameter settings of the individual robotic learning process. In particular, we showed that parameter settings resulting in a median performance experienced more benefits from robot-to-robot learning than parameter setting that already gave a high performance for one robot. As a consequence, research in robot-to-robot learning must consider the quality of the used parameter settings, as they can drastically impact the conclusion.

While this result explains the difference in observations in the current literature, we discovered an additional benefit of robot-to-robot learning that we investigate further in this paper. We show that robot-to-robot learning can reduce the sensitivity of the learning process to the choice of parameters for the individual learning process in two ways. First, robot-to-robot learning can reduce the number of bad performing individuals in the population within one run. Second, robot-to-robot learning can increase the chance of having a high performing individual in the population over multiple runs.

We study robot-to-robot learning in the context of on-line evolutionary robotics. In terms of the taxonomy defined by Haasdijk et al. (2012), they are defined as *hybrid* systems where robots can adapt their controller individually, but can also exchange information. The field of evolutionary robotics originated in the late 1980s and aims to create robotic controllers with Evolutionary Algorithms (EAs). These algorithms are inspired by Darwin's theory of survival of the fittest. In nature, animals survive and procreate when they are more fit. Similarly, a robotic controller is tested by observing the behavior of the robot and is given a corresponding fitness measure. The higher the fitness, the more chance this controller has to procreate. Over generations, the quality of the controllers will improve and lead to robots that are capable of executing a predefined task properly. The robotic controller that we consider is a neural network. A neural network is a direct policy that maps the sensor inputs of the robot to actions. This mapping, consisting of nodes and connections between the nodes, are evolved with EAs.

This paper is structured as follows: In section 2 we explain our method to select parameter settings and we describe measurements that we use to observe the benefits ofsocial learning. This approach is independent of the system described in section 3. We continue with the experimental setup in section 4 and present the results in section 5. An in depth discussion of the results is given in section 6. Our concluding remarks are summarized in section 7.

## 2. Parameter Settings and Measurements

Our implementation of the individual learning process has 21 parameters. These parameters are related to the individual learning mechanism of one robot. Depending on the chosen learning mechanism, the number of parameters can vary. The approach described in this section is independent of the specific individual learning mechanism of the robot. The parameters, of which 2 are boolean and 19 continuous, are presented in Appendix. Every parameter could be assigned a low, middle or high value. A combination of all parameters with their corresponding values is called a *configuration*.

Testing all possible configurations would require too much computational power. Therefore, Design of Experiments (DoE) was used to create the configurations. DoE is an approach that creates a minimal number of configurations to test while preserving the possibility to perform statistical analysis on the data (Montgomery, 2009). The DoE^1^ gave, for the case at hand with 19 continuous and 2 boolean parameters, an experimental design of 50 different configurations.

The configurations provided by the DoE are ranked based on their quality. The quality of a configuration is defined as the median performance in the final generation. The performance of a generation is defined as the median fitness of theindividuals in that generation. Rank number 1 is the configuration resulting in the highest quality and rank number 50 is the configuration resulting in the lowest quality.

Using the median performance measure is the most suitable measure for online evolutionary roboticsfor two reasons. First, the distribution of the fitness within one run and over different runs is very skewed. Second, for on-line evolution, it is more important that all controllers perform well as the robots both learn and perform their task at the same time. Therefore, if we mention performance, either a generation or a configuration, we always refer to the median performance unless mentioned otherwise.

For the robot-to-robot learning experiments, we use the best and median configurations. To avoid confusion, note that this median is not the median of multiple data points but based on the rank of the parameter setting. The best configurations are defined as the 10 settings that lead to the highest quality (rank 1–10). The median configurations are defined as the settings resulting in a median quality (rank 21–30). How robot-to-robot learning is exactly implemented is explained in section 3.

For a detailed analysis of the effects of robot-to-robot learning, we also need more detailed measurements for performance and learning speed. The measures presented here are not new but they have not (all) been used to study the benefits of robot-to-robot learning.

For the performance we use the following measures:

**Success Rate (SR)** The SR is measured by the percentage of the 20 replicate runs that have a controller in the final generation with good fitness. Agood fitness is a fitness equal to or higher than 75% of the maximum observed fitness over all runs. The maximum observed fitness in all runs is 12, resulting in a good fitness of 9 or higher.
The Success Rate Ratio (SRR) is calculated by dividing the SR of the robot-to-robot learning experiment by the individual learning experiment. An SRR higher than 1 means that robot-to-robot learning results in more successful runs.
**Population Failure (PF)** The PF is measured by the median percentage of bad controllers in the final generation over the 20 replicate runs. A bad performing robot is a robot with a fitness equal to zero.
The Population Failure Ratio (PFR) is calculated by dividing the PF of the individual learning experiment by the robot-to-robot learning experiment. A PFR higher than 1 means that robot-to-robot learning results in fewer bad performing robots in the final generation.

Note that these two measures act on a different level. The SR is calculated over multiple experiments, while a PF can be calculated for everyexperiment individually. The SR results in one value while the PF results in a value per experiment, of which the median is taken.

For the learning speed we use a measure that isolates the learning speed from the performance level:

**Learning Speed (LS)** The LS is measured as the numeric integral of the median performance over time (in a number of generations) of the 20 replicate runs. The performance is normalized with respect to the maximum median observed performance over time. In other words, it is the surface underthe median performance curve divided by the surface of a rectangle enclosing the highest median performance over time.
The Learning Speed Increase (LSI) is calculated by subtracting the learning speed of the individual learning experiment from the learning speed of the robot-to-robot learning experiment. An LSI higher than 0 means that robot-to-robot learning results in faster learning.

## 3. System Description

Parts of the system described in this section were already presented in previous work Heinerman et al. (2017). However, we decided to recite parts to increase the readability of this paper.

### 3.1. Task, Robot, and Environment

**Figure 1:**
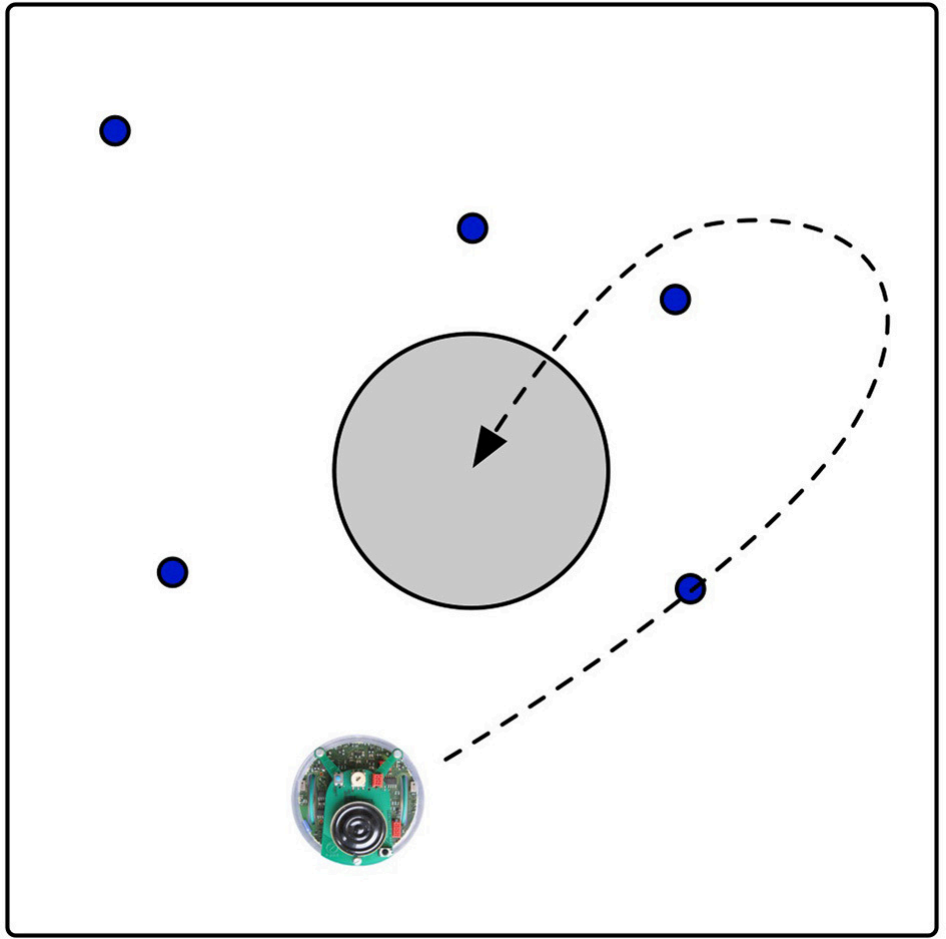
The environment with one robot searching for the blue pucks. The target location is indicated by the gray circle. The dashed line shows an example trajectory of a robot that picks up a puck to release at the target location.

The experiment we chose requires the robots to learn a foraging task. A foraging task requires the robot to collect pucks and bring them to the nest located in the center of the arena. The environment is a square arena as shown in Figure 1. Five pucks are randomly placed in the arena at the start of a run. Once a puck is brought to the nest, it is immediately moved to a random location in the environment. The fitness of each robot is equal to the number of pucks collected during a trial lasting 1,000 time steps.

The experiments are conducted in simulation using JBotEvolver (Duarte et al., 2014)^2^. JBotEvolver is a Java-based open-source, cross-platform framework for research and education in Evolutionary Robotics featuring a 2D differential-drive kinematics engine. The robots in our experiments simulate an e-puck robot. This robot is a small (7 cm) differential drive wheeled mobile robot equipped with 8 infrared proximity sensors. The range of these sensors is approximately 10% of the arena width. Additionally, the robots are equipped with the following task-specific sensors:

**Puck carrying sensor** Indicates if the robot is carrying a puck. The robot can carry one puck at a time;
**Puck sensor** Indicates the distance to the closest puck within the 45° perception cone of the sensor. The range of this sensor is approximately 60.
**Nest sensor** Indicates the distance to the nest if within the 45° perception cone of the sensor. The range of this sensor is approximately 60.

### 3.2. Controller and Individual Learning Mechanism

The robot's controller is an artificial neural network. The neural network has 11 input and two output nodes. The input nodes consist of 8 proximity sensors, a nest sensor, a puck sensor, and a puck carrying sensor; the output nodes provide the right and left motor speed. A neural network is a direct policy that maps the sensor inputs of the robot to actions. This mapping, consisting of nodes and connections between the nodes, are learned with an evolutionary algorithm.

Individual learning is implemented by an encapsulating, self-sufficient learning mechanism. The learning mechanism used in this paper is NEAT (Stanley and Miikkulainen, 2002). NEAT is an evolutionary algorithm that evolves both the topology and the connectivity of artificial neural networks. The initial population is composed of randomly generated feedforward neural networks without hidden layers. Every neural network is called an individual. Over time, nodes and connections can be added to the neural network, including the possibility of forming recurrent connections. All nodes have sigmoid activation functions.

Each robot possesses its own instance of NEAT. This means that each robot has a population of individuals, i.e., a set of controllers. These controllers are sequentially evaluated directly on the robot for 1,000 time steps. One time stamp is one sequence of the neural network where the sensor inputs are translated to an action. The fitness after these 1,000 time steps, the number of collected pucks, is stored. This fitness is used to select individuals for reproduction to create a new population.

The learning is conducted online, i.e., the robot is not relocated between the evaluations and each controller is tested starting from the location reached by the previous one. A consequence of online learning is that a controller can suffer from a bad initial position caused by a previous evaluation. Having to recover from a bad starting position can impact the fitness of the new controller in a negative way (Bredeche et al., 2009). In our set-up, the most common example of a bad starting position is being placed against a wall. To mitigate the negative effect of a bad starting position, we reposition the robot to a random location at the beginning of a controller evaluation when it was driving against the wall. This is to make sure that the learning does not stagnate because it is driving into the wall for a sequent number of evaluations and loses the diversity in the population of controllers.

### 3.3. Robot-to-Robot Learning Mechanism

When robot-to-robot learning is applied, every robot has its own arena as shown in Figure 1. Every robot is learning in an online fashion, while the robot is performing the task. Robot-to-robot learning is implemented as follows: first, the robots sequentially evaluate all controllers in their current population. Then, the robots exchange information. This means that the information is sent to another arena where another robot is located. This is different from having multiple smaller populations in one robot, because the position of the robot is a result of all evaluated controllers before. The robot compares the received controller?s fitness to that of its own worst controller. The new controller replaces the worst controller if it is better. The NEAT algorithm uses the updated list of controllers and fitness values to create the next generation.

As noted earlier, NEAT can modify the topology of the neural networks during evolution. Every structural modification in the network is identified by a unique innovation number to enable alignment of genomes for recombination purposes. When implementing NEAT with the possibility to exchange individuals as described for robot-to-robot learning, care must be taken to avoid conflicting innovation numbers. In our implementation, we keep track of a centralized global innovation database. If this would not be possible or desirable, one can use a distributed systems such as odNEAT (Silva et al., 2012) or Gene Clocks (Fernández Pérez et al., 2015)

Algorithm 1 summarizes the individual and robot-to-robot learning mechanism in pseudocode.

**Algorithm 1:** Pseudocode of the algorithm that runs on every robot

l initialise population of first generation (*P*_1_) with
individuals *i*_1_, …, *i*_*n*_
**while** current generation ≤ final generation
**for** every *i* in *P*
evaluate *i*
store *fitness* of *i*
sort the individuals based on fitness (*i*_1_ is best)
**if** robot-to-robot learning?
pick random other robot *R*
receive it's best individual *r*_1_
**if** *fitness*(*r*_1_)>*fitness*(*i*_*n*_)
*i*_*n*_ ← *r*_1_
create new population

## 4. Experimental Setup

We distinguish two different sets of experiments: the individual learning experiments, also called the *baseline experiments*, and the robot-to-robot learning experiments. Robot-to-robot learning experiments are performed with a group of 2 and 4 robots.

The baseline experiments are the 50 configurations given by the DoE. They are ranked based on the quality where after the best configurations (rank 1–10) and the median configurations (rank 21–30) are chosen to apply robot-to-robot learning too.

For all configurations, we use a fixed number of 20 k fitness evaluations. The total number of evaluations is the number of generations times the population size. Because the population size is a parameter in the configuration, we set the number of generations accordingly. As a result, 20 k evaluations correspond to 200 generations for a population size of 100, 334 generations for a population size of 60 and 1,000 generations for a population size of 20.

When robot-to-robot learning is applied, the robots have the same configuration as the one robot setup except for the population size. The population size for the robot-to-robot learning setup is the population size from the 1 robot setup divided by the number of robots to ensure the same number of evaluations per generation. Thus, if the original setup specifies a population size of 100, the robot-to-robot learning experiments use a population size of 50 and 25 for the 2 robots and 4 robot setup, respectively.

The robots operate in their own arena but they communicate across arenas. Consequently, the performance of the robot is only due to its own actions and not influenced by other robots in the same arena. Removing this inter-robot collision allows for a better comparison between the individual and the robot-to-robot learning experiments.

For all experiments, 20 replicate runs are performed with different random seeds.

## 5. Results

### 5.1. Baseline Experiments

**Figure 2:**
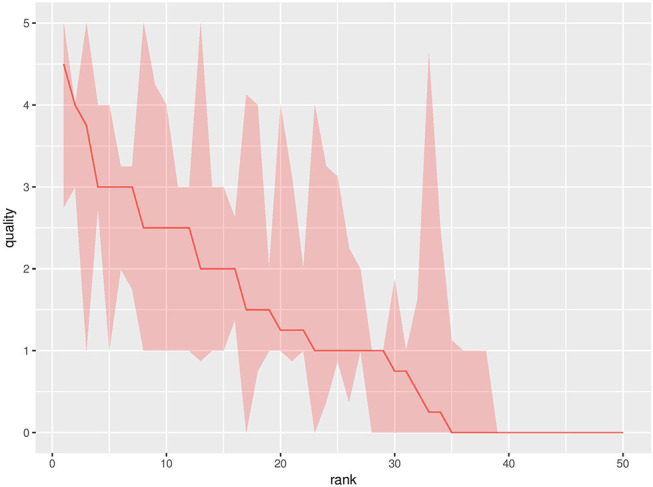
Median performance with an interquartile range of the baseline experiments for all DoE parameter settings at the final generation. The *y*-axis shows the performance, measured as the median number of collected pucks in the population. The *x*-axis shows the rank of the configuration. The results are compiled over 20 replicate runs.

The 50 configurations from the DoE are referred to as the baseline experiments. For the baseline experiments, there is only 1 robot, and it is learning individually. Figure 2 shows the quality and the interquartile range^3^ for all baseline experiments. If the median of two ranks is equal, the one with the highest value for the third quartile gets the better ranking. The quality differences between the configurations are clearly present: the lowest ranked setting has a quality of 0, while the highest ranked setting reached a quality of 4.5. The data in Figure 2 confirms that the configuration, e.g., the values of the parameters, significantly influence the quality of the individual learning result.

The data in Figure 2 shows the performance at the final generation for all 50 parameter configurations. It is not clear whether the performance for all configurations converged atthis point. To show that the performance has approximately converged at this point for most parameter configurations, we observe the increase in performance for all configurations from 95 to 100% of the evaluation budget. The median of this increase over the 50 configurations is 0 with an interquartile range of [0,0.1875], i.e., most of the 50 parameter configurations converged when they reach 95% of the evaluation budget. These statistics confirm that we used a sufficient number of evaluations for our experiments.

### 5.2. Robot-to-Robot Learning: Performance

**Figure 3:**
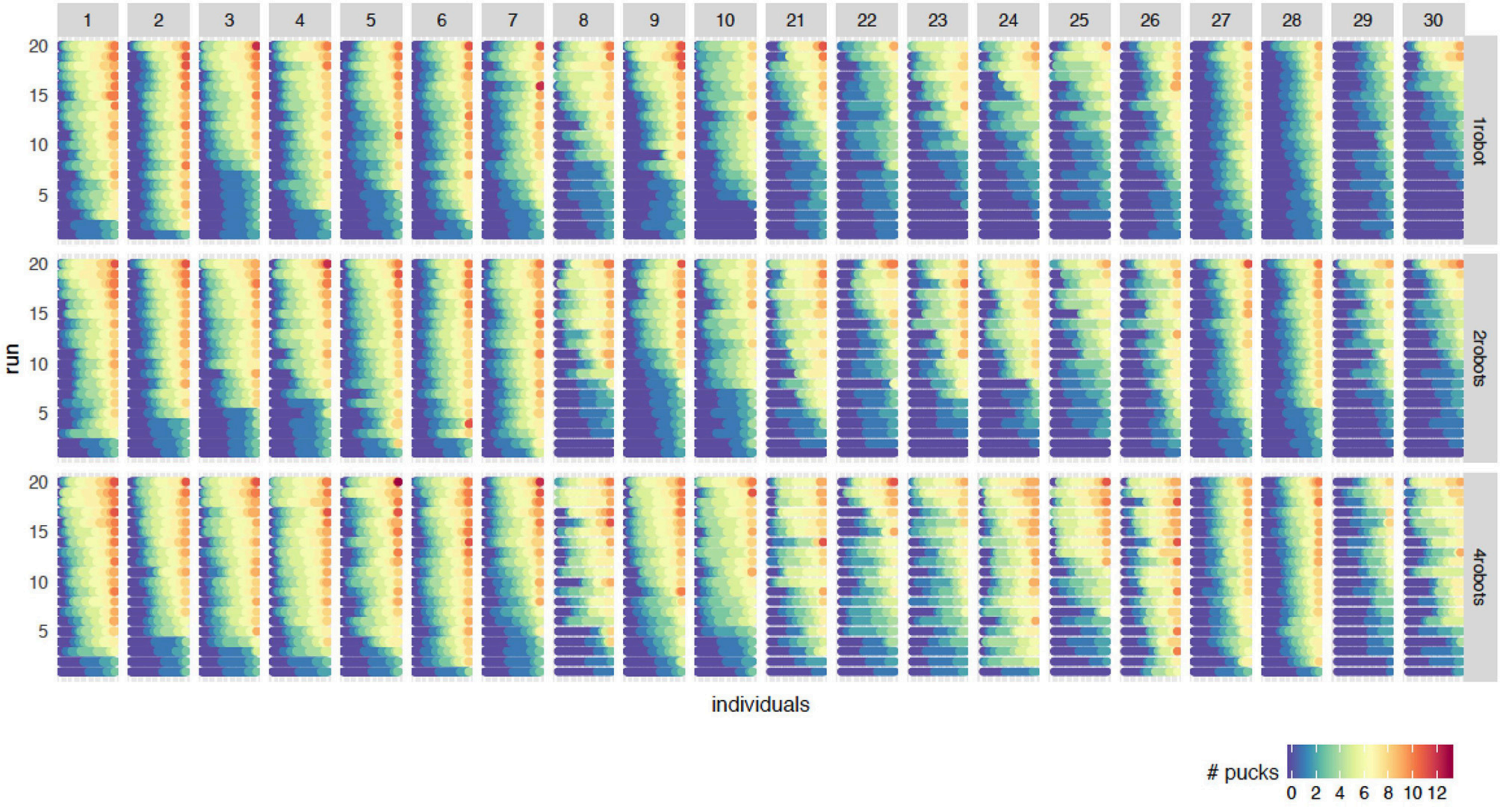
Fitness of the individuals at the final generation for all 20 runs for 1 robot **(top)**, 2 robot **(middle)** and 4 robots **(bottom)**. The columns refer to the rank of the configuration (rank 1–10 and 21–30). The 20 runs are sorted on the sum of the fitnesses of the individuals. Within one run, the individuals are sorted on the fitness of which the color reflects the value. When using multiple robots, the individuals of the final generation for all robots are combined and sorted on fitness. Therefore, every bar from blue to red actually consists of 100 dots where every dot represents one individual.

Figure 3 shows the fitness of the individuals at the final generation for all 20 independent runs and all robot-to-robot learning experiments. To explain this graph, we start at the first column (parameter configuration 1) and the first row of the 20 runs for 1 robot. This row, or bar, goes from blue to red, and actually consists of many dots, where each dot represents one individual in the final generation and the color represents the fitness of the individual. The bar consists of 100 dots, representing the fitness value of the individuals from all robots.

There are groups of 20 bars where each bar represents one run. The 20 runs are sorted on the sum of the fitnesses of the individuals. This block of 20 runs is shown for every combination of rank and the number of robots. The column refers to the rank of the configuration. Rank 1–10 (best configurations) and 21 to 30 (median configurations) are present. The rows refer to the number of robots. When using multiple robots, the individuals of the final generation for all robots are combined and sorted by fitness.

Figure 3 is an important graph to understand the effect of robot-to-robot learning. For the baseline experiments, we can observe the difference between configurations. Looking at the top row, the individual learning experiments, rank 1 to 10 shows more good performing individuals (red) and fewer bad performing individuals (blue) compared to rank 21–30. This is not a surprise since the configuration rank is based on the median performance of the individuals. In general, the runs with a high performing individual, have more red and yellow colored individuals than a run without a good performing individual. This results in an increase of the median performance measure.

However, an increase of median performing individuals is not a necessity for an increase in a number of successful runs. Looking at rank 28, we can clearly see an increase of performance of the best individuals within a run but not a decrease in the number of bad performing individuals. Therefore, we can conclude that the benefits of robot-to-robot learning highly depend on the particular configuration.

Due to the replacement of the worst performing individual for the robot-to-robot learning experiments, one might expect that the number of bad performing individuals decreases. This effect does happen for some parameter settings, such as setting 24, but not for all. The number of generations, and thus the number of times you receive the best controller, exceeds the size of the population. Especially for a population size of 20 (e.g., rank 21–25), there are 1,000 generations and thus 1,000 times to receive the best controller. However, in the final generation when the population size is 20, there are still bad performing robots. Therefore, we can say that simply replacing the worst controller with a betterone is not the reason that there are fewer badly performing controllers.

**Figure 4:**
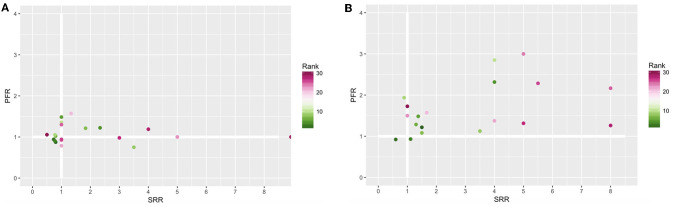
Population failure ratio (PFR) (*y*-axis) and success rate ratio (SRR) (*x*-axis) for 2 **(A)** and 4 **(B)** robots. The color presents the rank of the configuration. A value higher than 1 means that a benefit is observed when using robot-to-robot learning for that particular measure.

Figure 4 summarizes the information from Figure 3 by using the measures introduced in section 2. Figures 4A,B show the SRR and the PFR for the best and median configurations for the robot-to-robot learning experiment with 2 and 4 robots, respectively. A value higher than 1 means that a benefit due to robot-to-robot learning is observed for that particular measure.

From Figure 4 we can conclude that the benefits of robot-to-robot learning are more present for 4 robots than for 2 robots, indicated by fewer observations below the thicker white lines and the higher values for both measurements. Furthermore, we can observe that every configuration can have completely different benefits. An SRR of 8 can be observed for the 4 robot setup for rank 25 and 28 while the PFR increases slightly for rank 28 and a lot for rank 25. This is consistent with the observation of Figure 3.

Besides the influence of the configuration on the benefits of robot-to-robot learning, the group size also seems of influence. This can be further clarified when looking at rank 1 in depth. When applying robot-to-robot learning for 2 robots, the SR decreases from 50 to 40% while for 4 robots the SR increases from 50 to 75%.

Configuration 29 did not have any successful runs for the individual learning experiments. For both robot-to-robot learning experiments, with 2 and 4 robots, there were successful runs. Therefore, we can conclude that robot-to-robot learning can reach performance levels unreachable for the individual learning counterpart. This conclusion has also been presented in Jolley et al. (2016). Configuration 29 has been excluded from figure4 because the ratio cannot be calculated.

### 5.3. Robot-to-Robot Learning: Learning Speed

**Figure 5:**
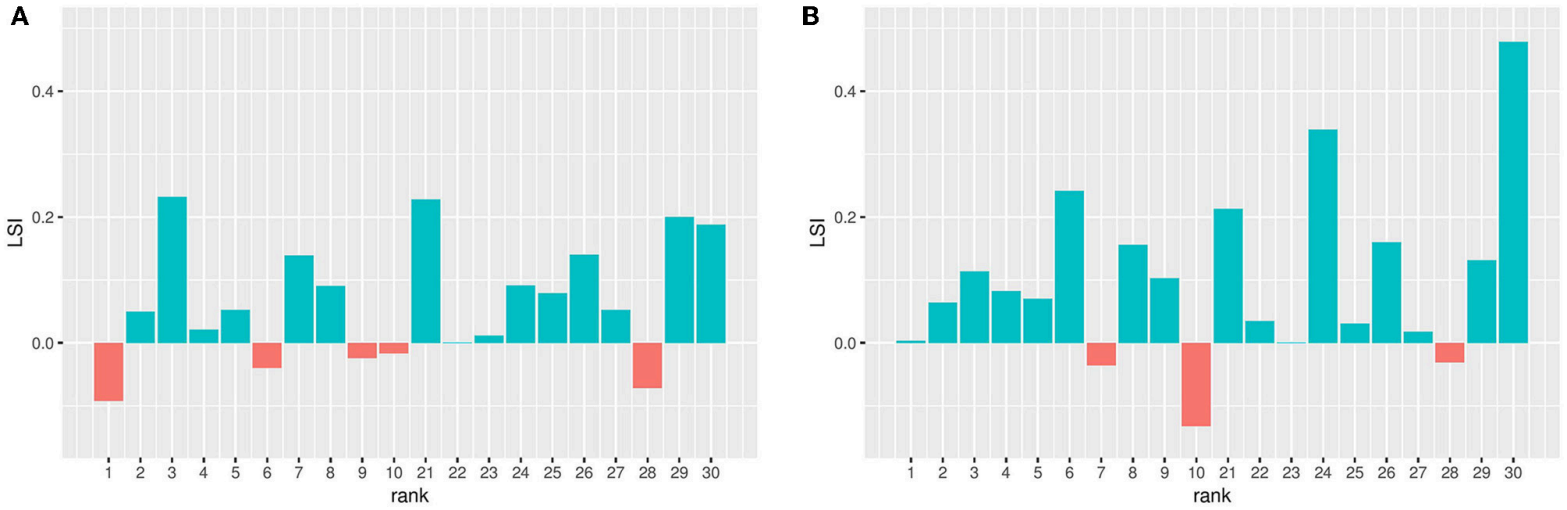
Learning speed increase (LSI) when using robot-to-robot learning with 2 **(A)** and 4 **(B)** robots. The LSI is calculated by subtraction the LS of one robot from the LS when using more robots. The LS is a numerical integral over the median performance over time and therefore one value. A value higher than 0 means that a benefit in learning speed is observed when using robot-to-robot learning.

Figures 5A,B show the LSI when using robot-to-robot learning for 2 and 4 robots, respectively. A value higher than 0 means that applying robot-to-robot learning results in faster learning compared to individual learning.

This graph shows an increased learning speed for most parameter setting for both 2 and 4 robots, indicated by the LSI values above 0. Robot-to-robot learning with 4 robots has a higher increase than robot-to-robot learning with 2 robots. The LSI for 2 robots correlates with the LSI for 4 robots [Pearson's *r*_(20)_ = 0.47, *p* = 0.0435]. This indicates that if there is a learning speed when using 2 robots, this is still also the case for 4 robots.

## 6. Discussion

In this paper, we performed a detailed analysis into the effects of robot-to-robot learning. In particular, we investigated the effect of the parameter settings, or configurations, of the individual learning mechanism on the benefits of robot-to-robot learning. The benefits of robot-to-robot learning were measured in three ways: (1) the success rate, which is the percentage of runs that have a good controller in the final generation (2) the population failure, which is the median of the percentage of bad individuals in the final generation and (3) the learning speed, which is the numerical integral of the median performance over time.

In previous work Heinerman et al. (2017) we concluded that configurations leading to a median quality for the individual learning process benefit more from robot-to-robot learning. In that work, we used the mean as the performance measurement. Due to the more specific measurements in this paper, we can conclude that the increase of performance of the median quality configurations is due to the increase in success rate. This can be seen in Figure 4 where the median parameter settings show a higher increase in the success rate. As a result, we can conclude that if the parameter settings are not optimal, individual learning needs more luck to have a good controller in the final generation. When we apply robot-to-robot learning this effect is mitigated with more successful runs as a result.

The parameter configurations impact the conclusion of the benefits of robot-to-robot learning. This might explain the contradicting observations in literature. However, we also have shown that robot-to-robot learning reduces the sensitivity to the choice of parameters. As a result, this might mean that even though different parameter setting has been used in literature which leads to different results, we could still compare the final performance in literature.

Additionally, from Figure 4 we conclude that robot-to-robot learning can reduce the number of bad performing individuals in the population. This number is important for online evolutionary robotics because the robots learn while executing a task, i.e., it is desirable that all robots have a good fitness instead of only one best.

This paper led to new insights into the effect of robot-to-robot learning. Robot-to-robot learning can reduce the sensitivity of the learning process to the choice of parameters in two ways: increase in the number of successful runs and a decrease of bad performing individuals. These two effects are similar to the effects desirable when tuning parameters. When tuning parameters the goal is to increase the average, median or maximum performance over all experiments. Preferably, these measures have a low variability. Increasing the success rate increases the average and possibly the maximum and median performance measures. Decreasing the population failure increases the average and potentially the median performance measure. Therefore, robot-to-robot learning can potentially reduce parameter tuning efforts.

We have seen that robot-to-robot learning can result in different benefits. Some parameter settings experience a small loss in performance and/or learning speed but most parameter settings experience a large gain in performance. While having observed the benefits in more detail, the question remains how the exchange of information results in these benefits. Due to the replacement of the worst performing individual for the robot-to-robot learning experiments, one might expect that the number of bad performing individuals decreases. But, we argued that this is not the reason due to the much higher number of controller exchanges compared to the population size. We believe it is an interplay between receiving new knowledge from other robots (resulting from a different search process) and less aggressive variation operators within one robot. This leads to more diverse quality solutions without increasing the effect of variation operators. However, we need extra data to confirm this hypothesis and this is left for future work.

Our specific implementation choice of robot-to-robot learning, has some similarities with parallel EAs and island models. These are commonly known to increase diversity which results in an increase in performance (Gordon and Whitley, 1993; Whitley et al., 1999; Alba and Tomassini, 2002; Park and Ryu, 2010). However, no research explains where the increase in diversity comes from and how this impacts the performance. Research in these areas mostly focusses on the decrease in runtime due to smaller population sizes (Alba and Tomassini, 2002). Measuring the runtime in evolutionary robotics is useless because the evaluation time of the robot is much larger than the computational effort. Additionally, the fitness function in evolutionary robotics is extremely stochastic. Although the results of both fields cannot be used to interpret each others work at the moment, we do believe that there are some common elements in parallel EAs, island models and robot-to-robot learning in evolutionary robotics. Especially, studying the effect of the number of parallels/robots on the diversity of the whole population is of interest to both fields. This will be investigated in future work.

Other interesting aspects of the benefits of robot-to-robot learning include the group size and task complexity. Our results indicate that using 4 robots results in more benefits than using 2 robots. This could potentially be due to the total number of controller exchanges between robots that varies depending on the number of generations. The influence of the frequency of controller exchanges on the speed of convergence in robot-to-robot learning can be taken into account when understanding the scalability of the group size.

Lastly, we should note that for the robot-to-robot learning experiments, we used the parameters of the 1 robot setup while these parameters might not be optimal for the multiple robot setups. A multiple robot setup itself can also benefit from parameter tuning, just as the parameters for the exchange of knowledge. In future research, we will study the effect of different parameter settings on the performance of the multi-robot setup. Additionally, one could change and or adapt the parameter settings per robot.

## 7. Conclusions

In this paper, we investigated the benefits of enabling robots to share knowledge with others. Existing literature in robot-to-robot learning typically compares individual learning with robot-to-robot learning for only one parameter setting. Our study extended this comparison by using 50 different parameter settings.

We showed that robot-to-robot learning can reduce the sensitivity of the learning process to the choice of parameters in two ways. First, robot-to-robot learning can reduce the number of bad performing individuals in the population. Second, robot-to-robot learning can increase the chance of having a successful run, where success is defined as the presence of a high performing individual. While some parameter settings experience a small decrease in performance, most parameter settings benefit greatly from robot-to-robot learning in terms of performance and/or learning speed.

Our results indicate that robot-to-robot learning is a powerful mechanism which leads to benefits in both performance and learning speed. Additionally, this paper showed the importance of an in-depth analysis to draw conclusions that are not possible with aggregated statistics. We hope to inspire others to use our proposed measurements for an in-depth analysis of components that have an impact on the benefits of robot-to-robot learning such as different tasks, the frequency of exchange, different group sizes and different environments.

## Author Contributions

JH contributed to the programming and running of the experiments, the visualization of the results, and the writing of the manuscript. EH contributed to the supervision of the experiments and the writing of the manuscript. AE contributed to the supervising the experiments and writing of the manuscript.

### Conflict of Interest Statement

The authors declare that the research was conducted in the absence of any commercial or financial relationships that could be construed as a potential conflict of interest.

## Footnotes

^1^created with the help of JMP software SAS Institute Inc, JMP, Version 11.1.0.

^2^The code is available on https://github.com/ci-group/ECAL_SocialLearning.

^3^The interquartile range fluctuates between configurations. This is due to the difference in population size. When the population size is small, the interquartile range is relatively large.

## Appendix

### Neat Parameters

The NEAT parameters with description and values (low, middle and high) for the DoE. Low, middle (mid) and high levels are used for numeric parameters are shown in the table below.

*Mutation and crossover parameters* _______________________________________

**Table:**

	Low	Mid	High
**p**_**Xover**_	0.05	0.20	0.35
chance to apply crossover			
**p**_**Mutation**_	0.1	0.25	0.4
chance to apply mutation on each node/link			
**p**_**WeightReplaced**_	0.0	0.25	0.5
chance to replace weight			
**max**_**Perturb**_	0.25	0.5	0.75
maximum allowed change on weight			
**p**_**AddLink**_	0.01	0.05	0.1
chance to add a link			
**p**_**AddNode**_	0.01	0.03	0.05
chance to add a node			
*Species parameters* _________________			
**species**_**Count**_	3	6	9
Maximum number of species.			
**max**_**SpeciesAge**_	6	18	30
maximum age of species			
**coeff**_**Excess**_	0.5	1.0	1.5
used for species compatibility score			
**coeff**_**Disjoint**_	0.5	1.0	1.5
used for species compatibility score			
**coeff**_**Weight**_	0.1	0.4	0.7
used for species compatibility score			
**threshold**	0.3	0.5	0.7
used for species compatibility score
**threshold**_**Change**_	0.01	0.1	0.2
used to change threshold value			
**species**_**AgeThreshold**_	0.7	0.75	0.8
percentage of age to count as old			
**species**_**YouthThreshold**_	0.2	0.25	0.3
percentage of age to count as young			
**age**_**Penalty**_	0.5	0.7	0.9
fitness multiplier for old individual			
**age**_**Boost**_	1.1	1.25	1.4
fitness multiplier for young individual			
*Other parameters* _________________			
**size**	20	60	100
population size of one robot			
**survival**_**Threshold**_	0.1	0.45	0.8
top % individuals that can be parents			
**copy**_**Best**_	TRUE	FALSE	
clone best individual previous generation			
**copy**_***BestEver***_	TRUE	FALSE	
clone best individual so far.			
